# The activation of BAFF/APRIL system in spleen and lymph nodes of *Plasmodium falciparum* infected patients

**DOI:** 10.1038/s41598-020-60763-3

**Published:** 2020-03-02

**Authors:** Wilanee Dechkhajorn, Surachet Benjathummarak, Supattra Glaharn, Urai Chaisri, Parnpen Viriyavejakul, Yaowapa Maneerat

**Affiliations:** 1 Department of Tropical Pathology, Faculty of Tropical Medicine, Mahidol University, 10400 Bangkok, Thailand; 2 Center of Excellence for Antibody Research, Faculty of Tropical Medicine, Mahidol University, 10400 Bangkok, Thailand

**Keywords:** Immunology, Molecular biology, Pathogenesis

## Abstract

Previous studies have reported activation of the B cell-activating factor (BAFF)/a proliferation-inducing ligand (APRIL) system in T independent immunity against malaria infection. *Plasmodium falciparum* (*P. falciparum*) infected animal model is not feasible. Therefore, little is known about the occurrence of BAFF/APRIL system and changes in falciparum lymphoid tissues. This study aimed to investigate the expression of BAFF/APRIL system components in lymphoid tissues from *P. falciparum* infected patients. Spleen and lymph node samples from 14 patients were collected at autopsy. Normal spleens and bacterially infected tonsils served as controls. The protein and/or mRNA expression of BAFF/APRIL and their cognate receptors, BAFF-R, TACI and BCMA, were determined by immunohistochemistry and RT-qPCR, respectively. The spleens of the patients exhibited significantly higher BAFF-R protein expression than normal spleens. Although without appropriate control, BCMA protein was markedly observed only in the lymph nodes. *BAFF* and *BCMA* mRNA levels were also significantly elevated in the spleen tissues of the patients compared with normal spleens. The overall BAFF-R protein levels in the lymphoid tissues of the patients correlated positively with parasitaemia. These findings are the first to confirm that BAFF/APRIL system activation in lymphoid tissues and is positively correlated with the parasitaemia levels in falciparum malaria.

## Introduction

The B cell-activating factor (BAFF)/a proliferation-inducing ligand (APRIL) system consists of BAFF, APRIL, and three cognate receptors expressed on B cells: B cell maturation antigen (BCMA), transmembrane activator and calcium modulator and cyclophilin ligand interactor (TACI)^1^, and BAFF receptor (BAFF-R)^2–4^. The BAFF/APRIL system plays multiple regulatory roles in the T cell independent (TI) immune response, including B cell activation, homeostasis, and survival^5^. APRIL binds with high affinity to both BCMA and TACI, while BAFF has lower affinity for these receptors and binds mainly to BAFF-R (reviewed in Dillon *et al*.^6^). Ligand binding triggers activation of diverse signalling pathways, including the mitogen-activated kinase and nuclear factor-κB pathways^7,8^ to induced immunoglobulin heavy chain class-switch recombination (CSR), and IgG and IgA production by B cells^5^. BAFF and APRIL are predominantly expressed by myeloid cells such as monocytes, macrophages, and dendritic cells^9^ and can be induced or upregulated by cytokines such as interferon-α and -γ, transforming growth factor-β, interleukin-4 and −10^2,^^10,11^.

Spleen and lymph nodes play pivotal roles in the host response to malaria. For example, the spleen eliminates Plasmodium parasitised red blood cells (PRBCs), and immune cells in both organs produce effector cytokines and specific anti-malaria antibodies that promote clearance of infection^12,13^. The BAFF/APRIL system plays well-characterised roles in numerous human disorders, including autoimmune diseases^14–19^, cancers^20^, and bacterial^21^, viral^22^, and fungal^23^ infections. Studies in humans^3^, and animal models^3,22,24,25^ have shown that BAFF/APRIL-mediated activation of B cells mainly occurs in bone marrow^26^ and secondary lymphoid tissues, particularly the spleen^3,24,25^, lymph nodes^24^, tonsils^3,27^, and gut-associated lymphoid tissue^21,22,28^. However, little is known about the interactions of components of the BAFF/APRIL system in lymphoid tissues from patients with *P. falciparum* malaria.

In this study, we investigated the expression of BAFF/APRIL pathway molecules in the spleen and lymph nodes from falciparum malaria patients. We examined BAFF, APRIL, BAFF-R, TACI, and BCMA levels and found that expression of several molecules was not only elevated during *P. falciparum* infection but also correlated positively with parasitaemia.

## Results

### Histopathological changes in lymphoid tissues from patients with falciparum malaria

**Figure 1:**
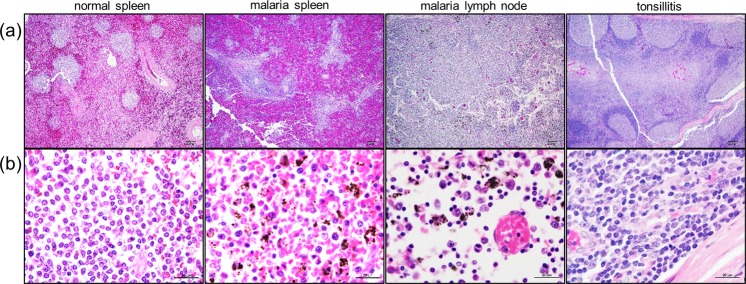
Histopathological staining of human lymphoid tissue sections. H&E staining of sections of normal spleen, spleen and lymph node from a patient with falciparum malaria, and bacterially infected tonsil at magnification ×40 (**a**) and ×1000 (**b**).

H&E-stained sections of spleens and lymph nodes were examined for *P. falciparum*-induced changes and the severity was scored using a semi-quantitative scale (− to ++++). Representative samples are shown in Fig. 1a,b, and more detailed descriptions are provided in Tables 1 and 2. Of the 14 patients, spleen and lymph node samples were obtained from 5 patients, spleen samples only were obtained from 5 patients, and lymph node samples only were obtained from 4 patients. Spleen sections generally exhibited severe changes typified by marked congestion and haemorrhage in both white and red pulps, congested red pulp, severely compressed white pulp with loss of architecture, and degenerated central arteries. Other pathologic changes included varying numbers of PRBCs and macrophages with engulfed malarial pigment (Table 1). In contrast, lymph node sections showed mild or moderate changes that included active lymphoid follicles with germinal centres, congestion, haemorrhage, and varying numbers of PRBCs and macrophages with engulfed malarial pigment (Table 2).

**Table 4:** Clinical data of *P. falciparum* infected patients (14 cases).

case no.	Diagnosis	Sex	Age	Days of fever	Parasitaemia /μl	Blood Hb (g/dl)	WBC count	Lymphocyte %	Monocyte %
1	CM	F	36	7	528,000	9.8	8,350	11	ND
2	NCM^a^	F	20	10	4,757	7.2	15,857	40	ND
3	CM	F	3	ND	655,220	ND	ND	ND	ND
4	CM	M	9	ND	2,982,000	ND	ND	ND	ND
5	NCM^a,b^	M	12	5	6,500	10.4	13,446	20	3
6	CM	M	24	8	2,242,800	8.6	22,462	12	1
7	CM	M	28	ND	21,300	7.3	23,926	18	4
8	CM	M	15	4	180,600	12	18,350	22	2
9	NCM^a,b^	M	19	5	412,500	10	10,200	30	5
10	CM	M	42	ND	493,200	8.4	7,950	34	2
11	NCM^c^	M	23	ND	34,500	5.1	10,650	40	1
12	CM	M	54	6	294,840	10	19,700	20	3
13	NCM^d^	M	15	5	132,600	11.4	30,400	9	3
14	CM	M	25	7	815,500	6.08	15,000	41	3

Note: ND; no data, M; male, F; female.

CM; cerebral malaria patients; *P. falciparum* infected patients died acutely of cerebral malaria whilst in coma and had impaired consciousness as their defining clinical examination criteria for diagnosis of CM according to WHO guideline (year 1991)^49^. In addition, the histological finding of parasitised RBCs (PRBCs) present in the brain tissues of CM cases.

NCM; non-cerebral malaria patients; *P. falciparum* infected patients died of other complications including ^a^pneumonia, ^b^septicaemia, ^c^acute renal failure, and/or ^d^severe anaemia.

**Table 1:** Histopathological characteristics of spleen tissue changes in *P. falciparum* infected patients (10 cases).

Case no.	Diagnosis	Gross description	Histopathologic changes				
			Changes in white pulps		Changes in red pulps		
			Compressed by red pulp	Central arteries show thickening wall with pink material	appearance	Macrophage with malarial pigment	PRBC (%)
1	CM	Marked congestion, mahogany, mushy	++	+/++	marked congestion	++++	30
3	CM	Mahogany-red and mushy	++	+	congestion, marked ghost cells of PRBC	+/++	90
4	CM	ND	++++	+	congestion	++++	80-90
6	CM	ND	++++	+	congestion, marked ghost cells of PRBC	++++	80-90
8	CM	ND	++++	+	congestion	++++	30
9	NCM	ND	+++	++	congestion, marked ghost cells of PRBC	++++	<10
10	CM	ND	+	++	marked congestion	++++	20
11	NCM	ND	+++	++	marked congestion	++++	<10
12	CM	ND	+ with normal architecture	− (normal)	Congestion, numerous ghost cells	+	80
13	NCM	ND	+++	+	Congestion, marked ghost cells of PRBC	++	5 and some plasma cells

Note: CM; cerebral malaria, NCM; non-cerebral malaria, ND; no data, PRBC; parasitised red blood cells.

### *BAFF-R*, *TACI*, and *BCMA* protein expression in lymphoid tissues from patients with falciparum malaria

**Figure 2:**
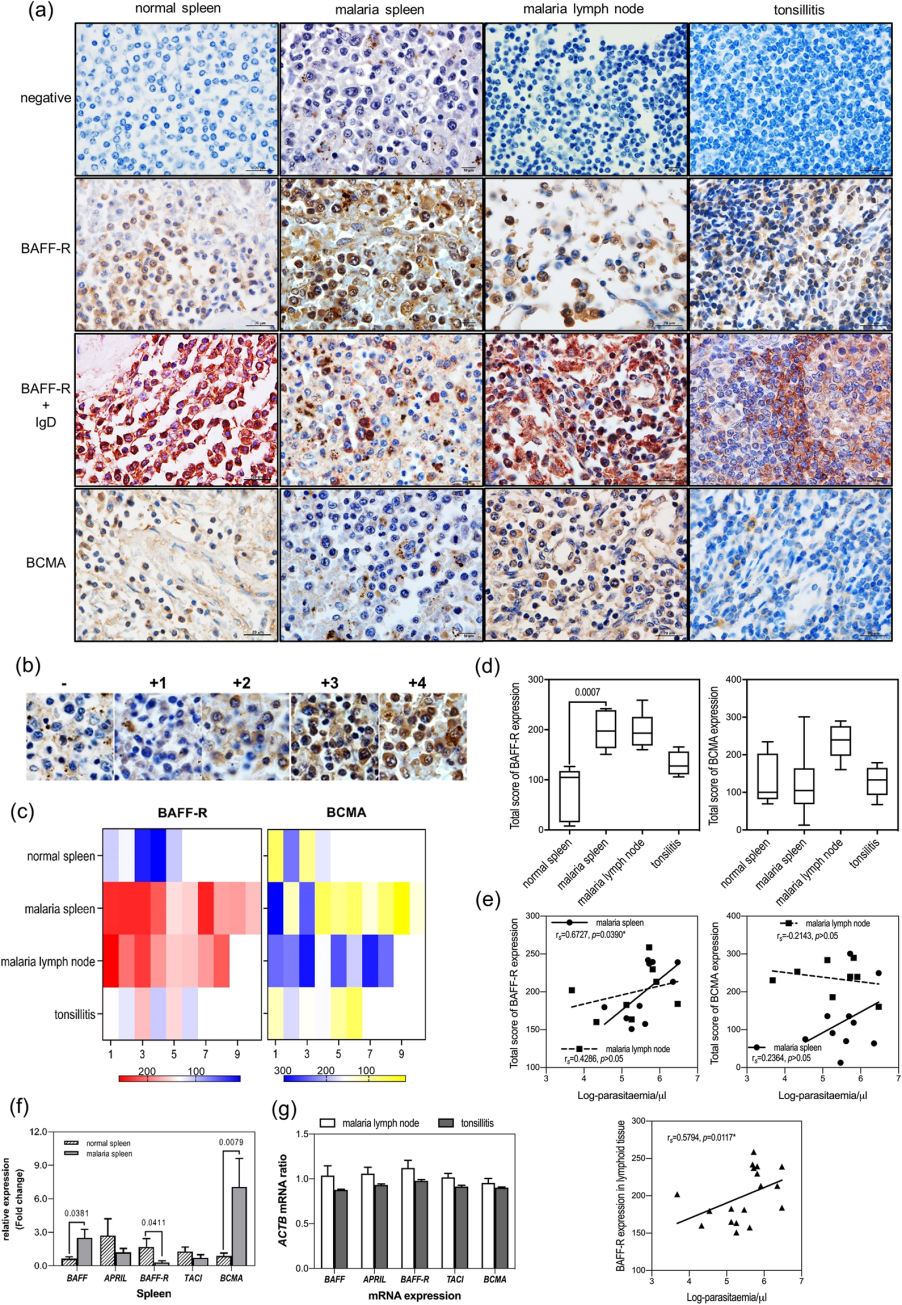
Expression of BAFF/APRIL and the cognate receptors in human lymphoid tissue sections. (**a**) Immunohistochemical staining of BAFF-R and BCMA in sections of normal spleen, spleen and lymph node from a patient with falciparum malaria, and bacterially infected tonsil at magnification ×1000. Negative = staining without primary antibody; single staining for BAFF-R or BCMA is brown; double staining for BAFF-R is brown in B cells (IgD+) is red. (**b,c**) Semi-quantification of immunohistochemical staining of BAFF-R and BCMA; (**b**) Representative images of spleen tissue sections from a patient with falciparum malaria stained with immunoperoxidase and DAB for BAFF-R showing intensity scores ranging from negative (−) to strongly positive (+4); (**c**) Heat maps showing intensity of total score immunoreactivity (the percentage of B cells with positive immunoreactivity × the intensity score) for BAFF-R (left panel) and BCMA (right panel); (**d**) Box and whisker plots showing total scores (percentage of B cells with positive staining × intensity score) for BAFF-R (left panel) and BCMA (right panel) expression in the indicated lymphoid tissue sections. Horizontal bar, box edges, and whiskers represent the median, the first/third quartiles, and the min/max values, respectively; (**e**) Correlations between parasitaemia and total staining scores for BAFF-R in spleen (left panel) or in both lymphoid tissues (lower panel), or BCMA in spleen of the malaria patients (right panel) using Spearman’s rank correlation test with a significance value of *p* < 0.05. (**f**) and (**g**) RT-qPCR analysis of *BAFF*, *APRIL*, *BAFF-R*, *TACI*, and *BCMA* mRNA levels in (**f**) spleens (fold change), and (**g**) lymph nodes (*ACTB* mRNA ratio) from patients with falciparum malaria or bacterially infected tonsil (positive control). Data were normalized to *ACTB* (β-actin) mRNA in the same sample. The results in the spleen of falciparum malaria patients are presented as the fold change in expression relative to the levels in the normal spleen samples using the 2^−(ΔΔCt)^ method^32^. The results for the lymph node tissues of the patients and tonsillitis were represented as *ACTB* mRNA ratio^29^.

The specificity of immunostaining for BAFF-R, TACI, and BCMA was confirmed using appropriate negative and positive controls, including the omission of primary antibodies, and staining of bacterially infected tonsil sections (Fig. 2a) or cultured Raji cells (data not shown), respectively. Representative images of sections stained for BAFF-R, BCMA, and BAFF-R + IgD in sections of normal spleen, spleen and lymph nodes from falciparum malaria patients, and bacterially infected tonsils are shown in Fig. 2a. The intensity of staining is shown in Fig. 2b. Receptor expression levels were evaluated on a semi-quantitative scale (0–400) (Fig. 2c) that incorporated both the intensity of staining and the percentage of cells with positive staining.

Total score of BAFF-R expression was significantly higher in spleen from falciparum malaria patients (200.58 ± 3.74) compared with normal spleen (73.82 ± 10.91). BCMA expression was markedly elevated in the lymph nodes (235.31 ± 5.54), but not the spleens, of falciparum malaria patients (Fig. 2d and Table 3). Figure 2e shows that the BAFF-R expression scores in the spleens of falciparum malaria patients correlated significantly with parasitaemia (*r*_*s*_ = 0.6727, *p* = 0.039) but not the BCMA expression scores of both lymphoid tissues. In addition, overall BAFF-R protein levels in these lymphoid tissues of patients correlated positively with parasitaemia (*r*_*s*_ = 0.5794, *p* = 0.0117). TACI expression was not detected in sections of the spleen or lymph nodes of falciparum malaria patients, normal spleen, or infected tonsil (data not shown).

**Table 2:** Histopathological characteristics of lymph node tissue changes in *P. falciparum* infected patients (9 cases).

Case no.	Diagnosis	Gross description	Histopathologic changes
1	CM	Not remarkable	Not active lymphoid follicles, not remarkable germinal center, no congestion, <1% PRBC
2	NCM	ND	Not remarkable except congestion, rare PRBC
3	CM	Enlargement	Not active lymphoid follicles, not remarkable germinal center, no congestion, some malarial pigments in macrophages
4	CM	Not remarkable, no hemorrhage	Not remarkable with normal architecture
5	CM	ND	Marked congestion and hemorrhage (++++) in cortex and subscapular space, no architecture of lymphoid follicles, numerous PRBC
7	CM	Jaundice	Not remarkable, few PRBC observed some malarial pigment in macrophage and some ghost PRBC
8	CM	ND	Marked congestion and hemorrhage (++++) in cortex and subscapular space, PRBC < 1%
13	NCM	ND	Moderate congestion and hemorrhage (++), lymphoid follicles are notable
14	CM, ARF, ARDS	Not remarkable	Congestion, not many PRBC in vascular space

Note: CM; cerebral malaria, NCM; non-cerebral malaria, ARF; acute renal failure, ARDS; acute respiratory distress syndrome, ND; no data, PRBC; parasitised red blood cells.

Comparing CM and NCM cases, the expression of BAFF-R and BCMA in the spleens or lymph nodes was not significantly different while the parasitaemia level in CM was significantly higher than NCM cases (Supplementary Table 1).

### BAFF-R, TACI, and BCMA mRNA expression in lymphoid tissues from patients with falciparum malaria

We performed RT-qPCR analysis to measure mRNA levels of the BAFF/APRIL system components in spleen (Fig. 2f), lymph node, and tonsil samples (Fig. 2g). *BAFF* mRNA expression in the spleens of patients with falciparum malaria was significantly higher than normal spleens (*p* = 0.0381). In contrast, *APRIL* mRNA expression tended to downregulate in the spleens of falciparum malaria patients compared with normal spleens (*p* > 0.05). *BAFF-R* mRNA expression (fold change) was significantly downregulated in the spleens of falciparum malaria patients (*p* = 0.0411), whereas *BCMA* mRNA expression was significantly upregulated only in the spleens of the patients (*p* = 0.0079) (Fig. 2f). Without comparison with normal relevant tissues, expressions of *BAFF, APRIL* mRNA and their receptor encoded mRNA in the lymph nodes of the patients were represented as *ACTB* mRNA ratios^29^ (Fig. 2g). The corresponding mRNA expressions in tonsillitis were positive control for RT-qPCR technique (Fig. 2g).

## Discussion

Polyclonal B cell activation and alterations in the composition of the B cell compartment are characteristic features of the host response to *P. falciparum* infection^30,31^. In the present study, we are the first to explore the possibility that the spleen and lymph nodes may be important sites of activation of B cells through the BAFF/APRIL signalling system during *P. falciparum* infection.

We previously demonstrated the TI production of HZ-specific IgG by human B cells co-cultured with monocytes, which was mediated through activation of the BAFF/APRIL system^32,33^. A number of previous studies have also reported activation of the BAFF/APRIL system by malaria infection; these include studies of patients with *P. falciparum* malaria^34–37^, patients with *P. vivax* malaria^36^ mice with *P. yoelii* malaria^38,39^ and *in vitro* studies of B cells stimulated by *P. falciparum*-derived antigens^33,40^. Muehlenbachs *et al*. investigated that expression of genes encoding IgG, IgM, CXCL13 (a B cell chemoattractant), and BAFF was increased in Tanzanian placental malaria^34^. Plasma BAFF levels were found to be increased and reflected disease severity in Kenyan children during acute falciparum malaria^35^. In that study, dynamic changes in B cell expression of BAFF-R, BCMA, and TACI were detected during the acute malaria phase and a 16-week follow-up period^35^. An earlier study found that infection of malaria-naïve humans with *P. falciparum* sporozoites resulted in increased BAFF expression on activated monocytes and dendritic cells, high plasma BAFF levels, and low BAFF-R expression on B cells during the acute phase of the challenge^37^.

The BAFF/APRIL system activation has previously been examined in a study of Brazilian patients infected with *P. falciparum* and *P. vivax*^36^. During the acute phase of infection, both species increased plasma APRIL levels whereas only *P. falciparum* resulted in elevated plasma BAFF levels^36^. The increase in soluble BAFF was in line with reports from other groups^35,37^. TACI expression was increased on T cells but not B cells during the acute phase of *P. vivax*-induced malaria^36^.

To our knowledge, the present study is the first to examine expression of BAFF/APRIL system components in secondary lymphoid tissue from falciparum malaria patients. We speculate that the elevated BAFF, APRIL, BAFF-R, TACI, and BCMA expressions in the lymphoid tissues reflect activation at the site of antibody production. The determination of these parameters to indicate occurrence of the BAFF/APRIL system activation was consistent with the previous studies in animal malaria^38,39^ and other human or animal diseases^3,21,26,27,41,42^ and cell cultures^3,43^.

In this study, we detected strong expression of BAFF-R protein in B cells in the spleen and lymph nodes from falciparum malaria patients, consistent with the elevated levels of *BAFF* mRNA in the same tissues. In contrast, *BAFF-R* mRNA was significantly downregulated in the tissues of falciparum malaria patients compared with normal spleen, which could be consistent with a high translation rate corresponding to the increase in protein expression. In this study, we could observe *TACI* mRNA in the positive control tonsillitis and malaria lymph node tissues (Fig. 2g). We did not find significant expression of TACI protein or mRNA in this study (Fig. 2d,f,g) despite the fact that TACI is known to be expressed on mature B cells and plasma cells and binds to both BAFF and APRIL^17^. TACI stimulates differentiation of plasma cells in response to TI-2 antigens^44^. Our failure to examine TACI protein expression was consistent with a previous study that TACI receptor constitutively released from activated B cells which produce soluble decoy receptors^45^.

We detected high expression of BCMA in the B cells of the lymph nodes of falciparum malaria patients, which could be explained by differing transcriptional and/or translational regulation in the two organs. *APRIL* mRNA, in contrast to BAFF, showed a trend towards downregulation in the spleens of falciparum malaria patients compared with normal spleens, whereas *BCMA* mRNA expression was significantly upregulated. APRIL is an important signalling molecule for the survival of plasma cells, which express BCMA^46^. Experiments *in vitro* suggest that plasma cells are more dependent on BAFF in the early stages of differentiation, whereas their long-term survival requires APRIL^47,48^. The reason for the observed trend of reduced *APRIL* mRNA levels is unclear. In our previous study^32^, we observed increased expression of APRIL, BAFF, BAFF-R, TACI, and BCMA in HZ-stimulated monocyte and B cell co-cultures. Therefore, our previous knowledge might contribute that APRIL is also play roles in falciparum malaria.

In addition to its role in the maturation of splenic B cells, BAFF-R is the major mediator of BAFF-dependent co-stimulatory responses in peripheral B and T cells. An earlier study of lymphoid follicles in tonsils revealed an important role for BAFF in the differentiation of germinal centre B cells, and showed that BAFF-R, TACI, and BCMA are differentially modulated during the differentiation of germinal centre B cells to plasma cells^42^.

In this study, we found significant positive correlations between the amount of parasitaemia and BAFF-R protein expression in the lymphoid tissues (spleen and lymph nodes) of falciparum malaria patients (*r*_*s*_ = 0.5794, *p* = 0.0117). Similarly, an earlier study in patients with falciparum placental malaria found that *BAFF* mRNA was significantly associated with the degree of malarial pigment engulfment by intervillous macrophages^34^.

The present study has some limitations. First, the sample size of spleen and lymph node tissues was small, due in large part to the rarity of autopsies in cases of falciparum malaria. Second, our results reflect the protein/mRNA expression pattern at the time of autopsy, and immunostaining of FFPE autopsied tissue may not accurately represent features of the BAFF/APRIL system in living patients. Third, we lacked normal lymph nodes for comparing changes of each variable expressed in the malaria lymph node tissues. In this study, tonsillitis tissues were only used as positive control for the expression of BAFF/APRIL system and immunostaining of BAFF-R, TACI and BCMA^3^. Fourth, unlike studies with animal models of malaria, we cannot manipulate the BAFF/APRIL system with agonists, antagonists, or blocking specific antibodies in patient tissues.

In conclusion, our results are the first to demonstrate that BAFF/APRIL-mediated B cell activation in the spleen and lymph nodes may play an important role in the immune response to *P. falciparum* infection.

## Methods

### Experimental design

The expression of BAFF/APRIL pathway molecules including BAFF, APRIL and the cognate B cell receptors in the spleen and lymph nodes from falciparum malaria patients were determined. The methods were carried out in accordance with the relevant guidelines and regulations. Approval for this study was obtained from The Ethical Committee, Faculty of Tropical Medicine, Mahidol University, Thailand (MUTM 2017-051-01 and MUTM 2017-051-02). Informed consent was obtained from all participants and/or their legal guardians.

### Patients and sample collection

Specimens of spleen and/or mesenteric lymph nodes were collected at autopsy from fourteen *P. falciparum* infected patients who died at The Hospital for Tropical Diseases, Faculty of Tropical Medicine, Mahidol University, between 1973 and 1998. Nine cases died of acute cerebral malaria (CM) whilst in a coma, and five severe cases (non-cerebral malaria, NCM) died of other complications, including pneumonia, septicaemia, acute renal failure, and/or severe anaemia. All CM cases had impaired consciousness as their defining clinical examination criterion for diagnosis of CM according to the WHO guidelines (1991)^49^. In addition, there was a histological finding of parasitized RBCs (PRBCs) in the brain tissues of CM cases. Samples of both spleen and lymph nodes were obtained from five patients, spleen only from five patients, and lymph nodes only from four patients. Normal spleen samples were obtained from five individuals who died from trauma due to traffic accidents between 1996 and 1999. Samples of bacterially infected tonsils were obtained from six individuals. The overall histopathologic changes of the tonsillitis sections stained with haematoxylin & eosin included noticeable congestion and haemorrhage in some areas, and most lymphoid nodules showed activation with an obvious germinal centre. A thickening epithelium was covered with fibrin, necrotic and pus cells, and a number of neutrophils. The clinicopathological characteristics of the fourteen patients are summarised in Table 4.

**Table 3:** Total score of BAFF-R and BCMA expressions in normal spleen, malaria spleen and lymph nodes, and tonsillitis tissues.

Tissue		Total score (mean ± SEM)	
		BAFF-R	BCMA
normal spleen	n = 5	73.82 ± 10.91	142.40 ± 18.84
malaria spleen	n = 10	200.58 ± 3.74*	125.30 ± 8.80
malaria lymph node	n = 9	199.30 ± 4.22	235.31 ± 5.54
tonsillitis	n = 6	132.19 ± 3.98	129.18 ± 6.91

Note: *Total score of BAFF-R pro tein expression was significant different between malaria and normal spleens using Mann-Whitney U Test. The results were considered statistically significant at the 95% confidence interval (*p* < 0.05).

### Tissue preparation and histopathological examination

Formalin-fixed, paraffin-embedded (FFPE) tissues of all samples were sectioned (4 μm thick). Standard histological study with Mayer’s haematoxylin and eosin (H&E) was conducted under light microscopy (BX41; Olympus, Japan). *P. falciparum*-induced histopathological changes in spleen and lymph node sections were categorised based on the severity of gross changes in tissue architecture, alterations to red and white pulp, macrophage infiltration, and PRBC abundance: grade 0, no visible histopathological changes; 1, minimal scattered changes; 2, moderate changes; 3, moderate to severe changes; and 4, severe histopathological changes. As examples, grade 4 changes in a spleen section might include marked congestion and haemorrhage in both white and red pulp, congestive red pulp, severely compressed white pulp, loss of architecture, degenerated central arteries, >80% PRBCs, and >90% macrophages with completely engulfed malarial pigment. In a lymph node section, grade 4 changes might include loss of architecture of the lymphoid follicle, marked congestion, severe haemorrhage, >80% PRBC in the cortex and subcapsular space, and abundant macrophages with completely engulfed malarial pigment.

### Immunohistochemistry

The primary antibodies were rabbit anti-human BAFF-R, goat anti-human TACI, rabbit anti-human BCMA (all from Thermo Fisher Scientific, Waltham, MA, USA), anti-human smooth muscle α-actin (1A4, DAKO, Glostrup, Denmark), and biotinylated anti-human IgD (SouthernBiotech, AL, USA). The secondary antibodies were biotinylated goat anti-IgG (Vector Laboratories, Inc., Burlingame, CA, USA), horseradish peroxidase (HRP)-conjugated rabbit anti-goat IgG (H + L) (Thermo Fisher Scientific). Antibody binding was developed using Vectastain ABC HRP Kit, NovaRed Peroxidase (HRP) Substrate Kit, and DAB Peroxidase (HRP) Kit (all from Vector Laboratories).

After microwave heating antigen-retrieval of tissue sections^50^, immunohistochemistry assay was conducted as described previously^3^ and examined by light microscopy (Olympus BX41).

Negative controls with primary antibodies omitted and positive controls (infected tonsil sections and the human B cell line (Raji) were processed in the same manner as above.

### Quantification of B cell receptor expression

For each tissue section, two independent observers (WD and YM) examined a total of 1000 IgD+ B cells in 20 random fields of view under oil immersion. Immunoreactivity for BAFF-R, TACI, and BCMA was evaluated on separate slides. Intensity of staining was scored semi-quantitatively using a 5-point scale: 0, negative; +1, scattered weakly positive; +2, weakly positive; +3, moderately positive; and +4, strongly positive (see Fig. 2b). The total score for each receptor was calculated as the percentage of B cells with positive immunoreactivity multiplied by the intensity score, with minimum and maximum scores of 0 and 400, respectively.

### Quantitative reverse-transcription PCR

To determine expression of mRNA encoding BAFF, APRIL, BAFF-R, TACI, BCMA, and β-actin, total RNA was extracted from FFPE tissues as described previously^51,52^. RT-qPCR were performed in duplicate^32,53^ using a Luna Universal One-Step RT-qPCR kit (New England Biolabs, MA, USA) and a CFX96TM Real-Time PCR Detection System (Bio-Rad Laboratories, CA, USA). Data were normalized to *ACTB* (β-actin) mRNA in the same sample. The results in the spleens of the falciparum malaria patients are presented as fold-change in expression relative to the levels in the normal spleen samples using the 2^−(ΔΔCt)^ method^32^. The results for the lymph nodes of the patients and tonsillitis were represented as *ACTB* mRNA ratio^29^.

### Statistical analysis

Non-parametric data were analysed using the Mann–Whitney U test for two groups or the Kruskal–Wallis test for more than two groups. Correlations between parameters were assessed using Spearman’s rank correlation test. All analyses using PASW Statistics 18 (SPSS Inc., Chicago, IL, USA). *p* < 0.05 was considered significant at the 95% confidence interval.

## Supplementary information

Supplementary information.

## Data Availability

Datasets generated and analyzed in this study are available from the corresponding author upon request.

## References

[1] Claudio E, Brown K, Park S, Wang H, Siebenlist U (2002). BAFF-induced NEMO-independent processing of NF-kappa B2 in maturing B cells. Nat Immunol.

[2] Nardelli B (2001). Synthesis and release of B-lymphocyte stimulator from myeloid cells. Blood.

[3] Ng LG (2004). B cell-activating factor belonging to the TNF family (BAFF)-R is the principal BAFF receptor facilitating BAFF costimulation of circulating T and B cells. J Immunol.

[4] Schneider P (2005). The role of APRIL and BAFF in lymphocyte activation. Curr Opin Immunol.

[5] Litinskiy MB (2002). DCs induce CD40-independent immunoglobulin class switching through BLyS and APRIL. Nat Immunol.

[6] Dillon SR, Gross JA, Ansell SM, Novak AJ (2006). An APRIL to remember: novel TNF ligands as therapeutic targets. Nat Rev Drug Discov.

[7] Hatzoglou A (2000). TNF receptor family member BCMA (B cell maturation) associates with TNF receptor-associated factor (TRAF) 1, TRAF2, and TRAF3 and activates NF-kappa B, elk-1, c-Jun N-terminal kinase, and p38 mitogen-activated protein kinase. J Immunol.

[8] Notas G (2012). APRIL binding to BCMA activates a JNK2-FOXO3-GADD45 pathway and induces a G2/M cell growth arrest in liver cells. J Immunol.

[9] Kimberley FC, Medema JP, Hahne M (2009). APRIL in B-cell malignancies and autoimmunity. Results Probl Cell Differ.

[10] Craxton A, Magaletti D, Ryan EJ, Clark EA (2003). Macrophage- and dendritic cell–dependent regulation of human B-cell proliferation requires the TNF family ligand BAFF. Blood.

[11] Ittah M (2008). Viruses induce high expression of BAFF by salivary gland epithelial cells through TLR- and type-I IFN-dependent and -independent pathways. Eur J Immunol.

[12] Buffet PA (2011). The pathogenesis of Plasmodium falciparum malaria in humans: insights from splenic physiology. Blood.

[13] Del Portillo HA (2012). The role of the spleen in malaria. Cell Microbiol.

[14] Chu VT, Enghard P, Riemekasten G, Berek C (2007). *In vitro* and *in vivo* activation induces BAFF and APRIL expression in B cells. J Immunol.

[15] Ju S (2006). Correlation of the expression levels of BLyS and its receptors mRNA in patients with systemic lupus erythematosus. Clin Biochem.

[16] Kang JS (2006). B cell-activating factor is a novel diagnosis parameter for asthma. Int Arch Allergy Immunol.

[17] Mackay F, Schneider P (2008). TACI, an enigmatic BAFF/APRIL receptor, with new unappreciated biochemical and biological properties. Cytokine Growth Factor Rev.

[18] Morel J (2009). Serum levels of tumour necrosis factor family members a proliferation-inducing ligand (APRIL) and B lymphocyte stimulator (BLyS) are inversely correlated in systemic lupus erythematosus. Ann Rheum Dis.

[19] Woo YJ (2011). Regulation of B cell activating factor (BAFF) receptor expression by NF-KappaB signaling in rheumatoid arthritis B cells. Exp Mol Med.

[20] Kern C (2004). Involvement of BAFF and APRIL in the resistance to apoptosis of B-CLL through an autocrine pathway. Blood.

[21] He B (2007). Intestinal bacteria trigger T cell-independent immunoglobulin A(2) class switching by inducing epithelial-cell secretion of the cytokine APRIL. Immunity.

[22] Yeramilli VA, Knight KL (2010). Requirement for BAFF and APRIL during B cell development in GALT. J Immunol.

[23] Xia R (2010). BLyS and APRIL expression in peripheral blood mononuclear cells of cryptococcal meningitis patients and their clinical significance. Clin Biochem.

[24] Vugmeyster Y (2006). A soluble BAFF antagonist, BR3-Fc, decreases peripheral blood B cells and lymphoid tissue marginal zone and follicular B cells in cynomolgus monkeys. Am J Pathol.

[25] Xu S, Lam KP (2001). B-cell maturation protein, which binds the tumor necrosis factor family members BAFF and APRIL, is dispensable for humoral immune responses. Mol Cell Biol.

[26] Maia S (2011). Aberrant expression of functional BAFF-system receptors by malignant B-cell precursors impacts leukemia cell survival. PLoS One.

[27] Xu W (2008). Viral double-stranded RNA triggers Ig class switching by activating upper respiratory mucosa B cells through an innate TLR3 pathway involving BAFF. J Immunol.

[28] Puga Irene, Cols Montserrat, Cerutti Andrea (2010). Innate signals in mucosal immunoglobulin class switching. Journal of Allergy and Clinical Immunology.

[29] Vaziri ND (2011). Nephrotic syndrome causes upregulation of HDL endocytic receptor and PDZK-1-dependent downregulation of HDL docking receptor. Nephrol Dial Transplant.

[30] Banic DM, Viana-Martins FS, De Souza JM, Peixoto TD, Daniel-Ribeiro C (1991). Polyclonal B-lymphocyte stimulation in human malaria and its association with ongoing parasitemia. Am J Trop Med Hyg.

[31] Portugal S, Pierce SK, Crompton PD (2013). Young lives lost as B cells falter: what we are learning about antibody responses in malaria. J Immunol.

[32] Dechkajorn W, Benjathummarak S, Kumsiri R, Maneerat Y (2018). The role of the BAFF/APRIL system in the T cell-independent specific response to blood stage Plasmodium falciparum hemozoin. Cytokine.

[33] Kumsiri R (2010). Blood stage Plasmodium falciparum antigens induce T cell independent immunoglobulin production via B cell activation factor of the TNF family (BAFF) pathway. Acta Trop.

[34] Muehlenbachs A, Fried M, Lachowitzer J, Mutabingwa TK, Duffy PE (2007). Genome-wide expression analysis of placental malaria reveals features of lymphoid neogenesis during chronic infection. J Immunol.

[35] Nduati E (2011). The plasma concentration of the B cell activating factor is increased in children with acute malaria. J Infect Dis.

[36] Pinna Raquel A., dos Santos Adriana C., Perce-da-Silva Daiana S., da Silva Luciene A., da Silva Rodrigo N. Rodrigues, Alves Marcelo R., Santos Fátima, de Oliveira Ferreira Joseli, Lima-Junior Josué C., Villa-Verde Déa M., De Luca Paula M., Carvalho-Pinto Carla E., Banic Dalma M. (2018). Correlation of APRIL with production of inflammatory cytokines during acute malaria in the Brazilian Amazon. Immunity, Inflammation and Disease.

[37] Scholzen A (2014). BAFF and BAFF receptor levels correlate with B cell subset activation and redistribution in controlled human malaria infection. J Immunol.

[38] Liu XQ (2012). Malaria infection alters the expression of B-cell activating factor resulting in diminished memory antibody responses and survival. Eur J Immunol.

[39] Parra M (2018). TACI Contributes to Plasmodium yoelii Host Resistance by Controlling T Follicular Helper Cell Response and Germinal Center Formation. Front Immunol.

[40] Donati D (2006). Increased B cell survival and preferential activation of the memory compartment by a malaria polyclonal B cell activator. J Immunol.

[41] Mohr E (2009). Dendritic cells and monocyte/macrophages that create the IL-6/APRIL-rich lymph node microenvironments where plasmablasts mature. J Immunol.

[42] Zhang X (2005). BAFF supports human B cell differentiation in the lymphoid follicles through distinct receptors. Int Immunol.

[43] Gui L (2016). IL-2, IL-4, IFN-gamma or TNF-alpha enhances BAFF-stimulated cell viability and survival by activating Erk1/2 and S6K1 pathways in neoplastic B-lymphoid cells. Cytokine.

[44] Mantchev GT, Cortesao CS, Rebrovich M, Cascalho M, Bram RJ (2007). TACI is required for efficient plasma cell differentiation in response to T-independent type 2 antigens. J Immunol.

[45] Hoffmann FS (2015). The immunoregulator soluble TACI is released by ADAM10 and reflects B cell activation in autoimmunity. J Immunol.

[46] Cassese G (2003). Plasma cell survival is mediated by synergistic effects of cytokines and adhesion-dependent signals. J Immunol.

[47] Avery DT (2003). BAFF selectively enhances the survival of plasmablasts generated from human memory B cells. J Clin Invest.

[48] Benson MJ, Elgueta R, Noelle RJ (2008). B cell survival: an unexpected mechanism of lymphocyte vitality. Immunol Cell Biol.

[49] World Health Organization. Management of Severe Malaria- a Practical Handbook (1st edition) (1991).

[50] Maneerat Y (2000). Inducible nitric oxide synthase expression is increased in the brain in fatal cerebral malaria. Histopathology.

[51] Ma Z, Lui WO, Fire A, Dadras SS (2009). Profiling and discovery of novel miRNAs from formalin-fixed, paraffin-embedded melanoma and nodal specimens. J Mol Diagn.

[52] Sharma M (2012). Ribonucleic acid extraction from archival formalin fixed paraffin embedded myocardial tissues for gene expression and pathogen detection. J Clin Lab Anal.

[53] Loseke S, Grage-Griebenow E, Wagner A, Gehlhar K, Bufe A (2003). Differential expression of IFN-alpha subtypes in human PBMC: evaluation of novel real-time PCR assays. J Immunol Methods.

