# Supplementary material for: The activation of BAFF/APRIL system in spleen and lymph nodes of Plasmodium falciparum infected patients
Source: Sci Rep. 2020 Mar 2;10:3865. doi: 10.1038/s41598-020-60763-3 (PMC7052189; doi:10.1038/s41598-020-60763-3)
Supplement: Supplementary file 1 — Supplementary information. [file 41598_2020_60763_MOESM1_ESM.docx]

**The activation of BAFF/APRIL system in spleen and lymph nodes of fatal *Plasmodium falciparum* infected patients**

**Wilanee Dechkhajorn^1^, Surachet Benjathummarak^2^, Supattra Glaharn^1^, Urai Chaisri^1^, Parnpen Viriyavejakul^1^, Yaowapa Maneerat^1^***

^1^Department of Tropical Pathology, Faculty of Tropical Medicine, Mahidol University, Bangkok, Thailand, 10400

^2^Center of Excellence for Antibody Research, Faculty of Tropical Medicine, Mahidol University, Bangkok, Thailand, 10400

^*^Corresponding author: Department of Tropical Pathology, Faculty of Tropical Medicine, Mahidol University, 420/6 Ratchawithi Road, BKK, Thailand 10400. Telephone: +662-306-9100 ext. 1620, Fax: +662-306-9184. Email: yaowapa.man@mahidol.ac.th

Conflict of interest statement: The authors declare that they have no competing interests.

**Supplementary Table 1** Comparison of variables between CM and NCM patients using Mann-Whitney *U* Test.

| **variables** | **mean ± SEM** | | ***p*-value*** |
| --- | --- | --- | --- |
|  | **CM (n=9)** | **NCM (n=5)** |  |
| parasitaemia | 912,606.67  ± 364,229.21 | 118,171.40  ± 77,191.88 | 0.0190* |
| BAFF-R (spleen) | 214.76 ± 13.49 | 167.49 ± 6.53 | 0.1167 |
| BAFF-R (lymph node) | 201.60 ± 15.99 | 192.39 ± 9.71 | 0.8571 |
| BCMA (spleen) | 138.91 ± 38.63 | 93.54 ± 21.16 | 0.8333 |
| BCMA (lymph node) | 228.02 ± 19.20 | 257.19 ± 26.68 | 0.8571 |

* The results were considered statistically significant at the 95% confidence interval (*p* < 0.05)
